# GeneLibrarian: an effective gene-information summarization and visualization system

**DOI:** 10.1186/1471-2105-7-392

**Published:** 2006-08-29

**Authors:** Jung-Hsien Chiang, Jyh-Wei Shin, Heng-Hui Liu, Chong-Liang Chin

**Affiliations:** 1 Department of Computer Science and Information Engineering, National Cheng Kung University, Tainan, Taiwan; 2 Department of Parasitology, College of Medicine, National Cheng Kung University, Tainan, Taiwan

## Abstract

**Background:**

Abundant information about gene products is stored in online searchable databases such as annotation or literature. To efficiently obtain and digest such information, there is a pressing need for automated information-summarization and functional-similarity clustering of genes.

**Results:**

We have developed a novel method for semantic measurement of annotation and integrated it with a biomedical literature summarization system to establish a platform, GeneLibrarian, to provide users well-organized information about any specific group of genes (*e.g*. one cluster of genes from a microarray chip) they might be interested in. The GeneLibrarian generates a summarized viewgraph of candidate genes for a user based on his/her preference and delivers the desired background information effectively to the user. The summarization technique involves optimizing the text mining algorithm and Gene Ontology-based clustering method to enable the discovery of gene relations.

**Conclusion:**

GeneLibrarian is a Java-based web application that automates the process of retrieving critical information from the literature and expanding the number of potential genes for further analysis. This study concentrates on providing well organized information to users and we believe that will be useful in their researches. GeneLibrarian is available on

## Background

Imagine the following situation. Your search engine at the NCBI site finds out that in addition to the 400 medical documents that match your query, another 400 are also relevant, but they are just one of the 44,000 genes at your favorite microarray chip. Imagine now that you have a sophisticated software that will automatically extract the most useful information from all the documents and summarize it for you in sentences so that you don't have to read the entire documents!

Abundant information about gene products is stored in online searchable databases such as annotation or literature. To efficiently obtain and digest such information, there is a pressing need for automated information-summarization and functional-similarity clustering of genes. A growing number of researchers have attempted to annotate gene products via controlled vocabularies in Gene Ontology (GO), given that gene ontologies are central to most biological processes and key footnotes of protein functions [1]. At the same time, current depictions of the relationships of cross-referencing are done manually, and cellular interactions and functional roles of molecules are not being captured in a single clear global snapshot, a hindrance to efficient knowledge discovery.

Some work has been done on discovering new and potentially meaningful relationships between medical concepts by searching and analyzing the annotation databases [2,3]. We believe it would be useful for biologists to have well-organized up-to-date information about their genes of interest when they want it. Therefore, the aim of our research is to offer researchers an electronic and self-generating reference-search system of functional associations, and to provide automatically updated summarized information embedded in PubMed abstracts for any given group of genes.

## Implementation

**Figure 1:**
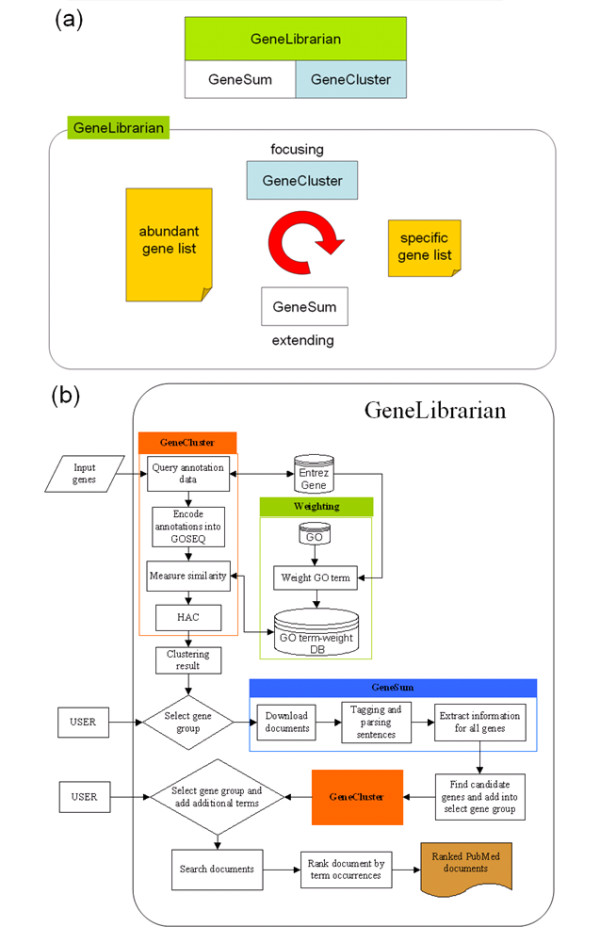
(a) GeneLibrarian was contructed based on GeneSum and GeneCluster. (b) System workflow of the GeneLibrarian.

In this study, we constructed two main modules in the GeneLibrarian system. The first one, GeneCluster, was developed to help users understand the functional distribution of a certain set of genes by visualizing the degree of semantic similarity between their GO annotations. The other one is a text mining-based gene information summarization module, which extracts useful information about gene products from PubMed abstracts, such as related genes, functions, and diseases. Figure 1 shows a schematic flow diagram of the method, which consists of two modules in the GeneLibrarian system: the GeneCluster and the GeneSum. GeneLibrarian integrates the applications of GeneCluster and GeneSum. GeneCluster is applied to provide a functional relationship graph of annotations in abundant gene list as reference which helps users to focus on functional related group of genes. GeneSum, moreover, extracts relevant information regarding the specific gene list that user selected from the result of GeneCluster. Cooperating with these two modules, GeneLibrarian facilitates users to refine the gene list and effectively collects relevant information as more as possible. In addition, GeneLibrarian provides an enhanced information retrieval agent, which submits queries to NCBI PubMed according to the combination of user specified keywords and selected genes and then displays results in ranked PMIDs by counting the appearance of user specified information.

### GeneCluster – visualization of functional relationship among genes

Brief descriptions of the GeneCluster follow. To quantify the degree of semantic similarity between GO annotations, we propose a novel sequence-alignment-based measurement to determine how similar two annotated concepts are. Because every GO term has a different biological meaning, it is pivotal to assign each term a weight that reflects its information content as well as the research activities. For each GO term t, the annotating frequency, p(t), is determined from the human genomic annotations of Entrez Gene [4]. This value indicates, as a percentage, how many genes each node or any of its children annotates. Here the weight of a GO term is defined by its information content:

*weight*(*t*) = -ln(*p*(*t*))       (1)

Such a strategy assigns lower weights to GO terms with more annotations and wider semantic meaning and that are closer to the root. Similarly, it assigns higher weights to GO terms with the opposite attributes. A path from a certain GO term toward the root of the ontology is treated as a sequence (GOSEQ). Suppose there are two such sequences, GOSEQi and GOSEQj, with lengths i and j, respectively. The similarity SSeq between them is defined as

SSeq(GOSEQi,GOSEQj)=∑k=1max⁡(i,j)S(Tki,Tkj)S(T1,T2)={weight(T1)T1=T2−(MaxP−PreMatch)T1≠T2     (2)
 MathType@MTEF@5@5@+=feaafiart1ev1aaatCvAUfKttLearuWrP9MDH5MBPbIqV92AaeXatLxBI9gBaebbnrfifHhDYfgasaacH8akY=wiFfYdH8Gipec8Eeeu0xXdbba9frFj0=OqFfea0dXdd9vqai=hGuQ8kuc9pgc9s8qqaq=dirpe0xb9q8qiLsFr0=vr0=vr0dc8meaabaqaciaacaGaaeqabaqabeGadaaakeaafaqabeGabaaabaGaem4uamLaem4uamLaemyzauMaemyCaeNaeiikaGIaee4raCKaee4ta8Kaee4uamLaeeyrauKaeeyuae1aaSbaaSqaaiabdMgaPbqabaGccqGGSaalcqqGhbWrcqqGpbWtcqqGtbWucqqGfbqrcqqGrbqudaWgaaWcbaGaemOAaOgabeaakiabcMcaPiabg2da9maaqadabaGaem4uamLaeiikaGIaemivaq1aa0baaSqaaiabdUgaRbqaaiabdMgaPbaakiabcYcaSiabdsfaunaaDaaaleaacqWGRbWAaeaacqWGQbGAaaGccqGGPaqkaSqaaiabdUgaRjabg2da9iabigdaXaqaaiGbc2gaTjabcggaHjabcIha4jabcIcaOiabdMgaPjabcYcaSiabdQgaQjabcMcaPaqdcqGHris5aaGcbaGaem4uamLaeiikaGIaemivaq1aaSbaaSqaaiabigdaXaqabaGccqGGSaalcqWGubavdaWgaaWcbaGaeGOmaidabeaakiabcMcaPiabg2da9maaceqabaqbaeqabiGaaaqaaiabdEha3jabdwgaLjabdMgaPjabdEgaNjabdIgaOjabdsha0jabcIcaOiabdsfaunaaBaaaleaacqaIXaqmaeqaaOGaeiykaKcabaGaemivaq1aaSbaaSqaaiabigdaXaqabaGccqGH9aqpcqWGubavdaWgaaWcbaGaeGOmaidabeaaaOqaaiabgkHiTiabcIcaOiabd2eanjabdggaHjabdIha4jabdcfaqjabgkHiTiabbcfaqjabbkhaYjabbwgaLjabb2eanjabbggaHjabbsha0jabbogaJjabbIgaOjabcMcaPaqaaiabdsfaunaaBaaaleaacqaIXaqmaeqaaOGaeyiyIKRaemivaq1aaSbaaSqaaiabikdaYaqabaaaaaGccaGL7baaaaGaaCzcaiaaxMaadaqadiqaaiabikdaYaGaayjkaiaawMcaaaaa@9886@

where *T*^*i *^and *T*^*j *^are GO terms in GOSEQi and GOSEQj, respectively. MaxP is the maximum penalty score, and PreMatch is the weight of the last matched term. This method is characterized by the penalty/reward schema in which mismatched GO terms receive fewer penalties, while matched ones receive more rewards as they move more deeply into the hierarchy when comparing two paths. This accurately reflects the semantic similarity within the GO structure. The similarity between GO terms ti and tj is defined as:

Sim(ti,tj)=max⁡GOSEQi∈ti,GOSEQj∈tjSSeq(GOSEQi,GOSEQj).     (3)
 MathType@MTEF@5@5@+=feaafiart1ev1aaatCvAUfKttLearuWrP9MDH5MBPbIqV92AaeXatLxBI9gBaebbnrfifHhDYfgasaacH8akY=wiFfYdH8Gipec8Eeeu0xXdbba9frFj0=OqFfea0dXdd9vqai=hGuQ8kuc9pgc9s8qqaq=dirpe0xb9q8qiLsFr0=vr0=vr0dc8meaabaqaciaacaGaaeqabaqabeGadaaakeaacqWGtbWucqWGPbqAcqWGTbqBcqGGOaakcqWG0baDdaWgaaWcbaGaemyAaKgabeaakiabcYcaSiabdsha0naaBaaaleaacqWGQbGAaeqaaOGaeiykaKIaeyypa0ZaaCbeaeaacyGGTbqBcqGGHbqycqGG4baEaSqaaiabdEeahjabd+eapjabdofatjabdweafjabdgfarnaaBaaameaacqWGPbqAaeqaaSGaeyicI4SaemiDaq3aaSbaaWqaaiabdMgaPbqabaWccqGGSaalcqWGhbWrcqWGpbWtcqWGtbWucqWGfbqrcqWGrbqudaWgaaadbaGaemOAaOgabeaaliabgIGiolabdsha0naaBaaameaacqWGQbGAaeqaaaWcbeaakiabdofatjabdofatjabdwgaLjabdghaXjabcIcaOiabdEeahjabd+eapjabdofatjabdweafjabdgfarnaaBaaaleaacqWGPbqAaeqaaOGaeiilaWIaem4raCKaem4ta8Kaem4uamLaemyrauKaemyuae1aaSbaaSqaaiabdQgaQbqabaGccqGGPaqkcqGGUaGlcaWLjaGaaCzcamaabmGabaGaeG4mamdacaGLOaGaayzkaaaaaa@71DF@

**Figure 2:**
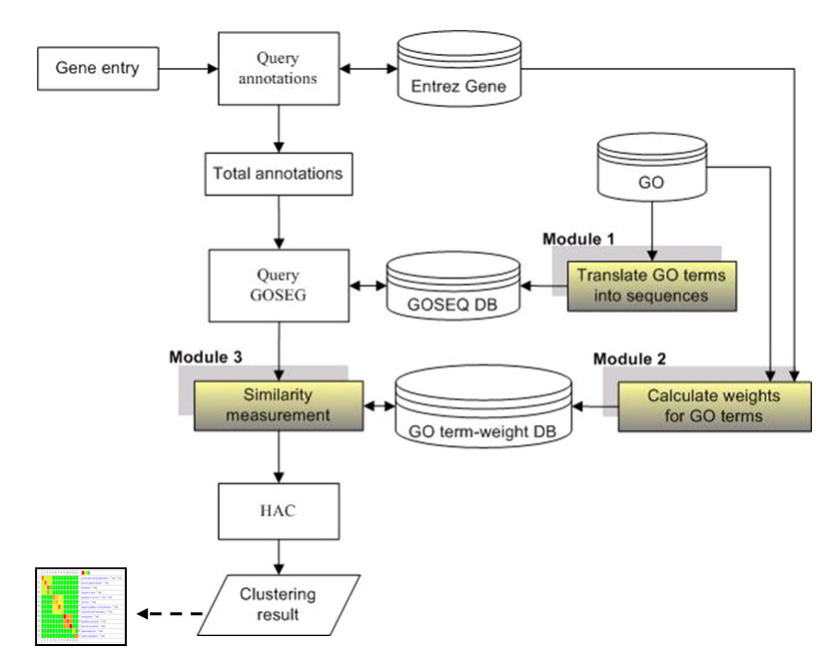
Schematic diagram of the GO-based genes clustering algorithm.

Based on this measurement, GO annotations of selected genes could be organized by applying the hierarchical agglomerative clustering (HAC) algorithm in hopes of appropriately grouping them according to the closeness of their functional annotations, as shown in Figure 2. We then exhibit the clustering results in a colorful 2D array in which hotter color indicates higher similarity, and vice versa.

### GeneSum – text mining-based summarization module

In addition, the GeneSum tackles the issues of literature information summarization. The algorithm of GeneSum proceeds as follows:

#### Step 1

Document Preprocessing. The purpose of the preprocessing step is to collect relevant documents and filter out those sentences without mentioning keywords according to a customized lexicon for later stages. Each sentence is regarded as a transaction and these candidate terms are items in transactions.

#### Step 2

**Figure 3:**
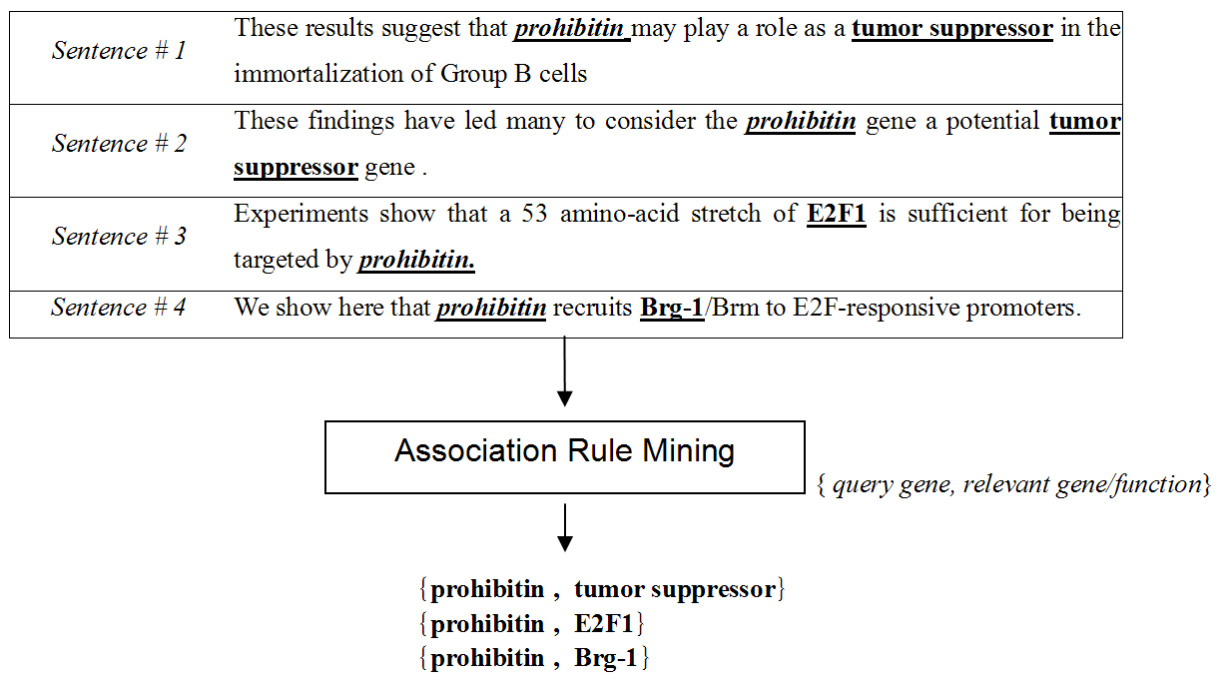
Example of the association rules mining for gene, function, and disease relations.

Large ItemSets Mining. After detecting those candidate terms, the Apriori association mining algorithm [5,6] is employed to find corresponding large items sets for summarization. Those large items are candidate genes or functions or diseases. Figure 3 shows an instance of mining candidate items from sentences. In order to confirm that large items mentioned in the same sentence are really relevant, sentences containing large items are then passed to next processing step.

#### Step 3

Sentence Structure Simplification. Large itemsets mining is a statistical method to identify candidate items which may be relevant. In order to confirm the accuracy of their relationships, the evidence in original sentences mentioning these large items should be extracted. But the complex structure of sentence is an obstacle for computer to extract the relationships of items. Therefore, we used natural language processing (NLP) technology such as part-of-speech (POS) tagging and phrase chunking to simplify the structure of candidate sentences in order to improve the accuracy of extraction of critical information for summarization. We use following three steps to achieve the goal:

1. POS tagging: Part-of-speech information is essential for GeneSum to analyze the sentences. Before further analysis, we employ Brill's POS tagger[7] to annotate text with part-of-speech.

2. Noun phrase chunking: In biomedical text many proper nouns are complex, such as "breast cancer" or "tumor supressor gene" etc, and their POS tags usually lead to confusion in information extraction. Here, we've developed chunking rules, shown in Table 1, to identify these proper nouns and reduce the complexity of sequence of POS tags.

3. Adjacent phrases merging: Sometime the desired information may be described as "<gene A> interacts with <gene B> and <gene C>". This sentence mentions two relationship: "<gene A> interacts with <gene B>" and "<gene A> interacts with <gene C>". In order to extract this kind of relationship, we first merged phrases connected by conjunction, such as and/or, and regarded them as a single noun tagged with NN. Thus, the sentence structure is further simplified and this benefits recognizing piece of text that does describes the desired relationships.

The simplified sequence of POS tags is the input of the finite-state-automata machine described below. Hence correct simplification of sentence structure will improve the accuracy of extracted information.

#### Step 4

Summary Generation. We designed a *9-state *finite-state-automata(FSA) machine[8] to recognize piece of text describing relationships of genes and functions and diseases according to the sentence structures, i.e. sequence of POS tags. In this step each set of candidate genes, functions and diseases obtained in step 2, and the sequence of POS tags of corresponding sentence are inputs, and outputs are those pieces of sentences that describe the relationships of candidate these items. The FSA is illustrated in Figure 3. The states are numbered from 1 to 9. State 4 and state 8 are terminal states, and the others are not. Transition from state to state is trigged by tags of four major classes: NN, VB, IN, CC. Tags not belonging to any of the four classes will be ignored, such as "Determiner" tag, DT. Once the FSA encounters a tag belonging to one of the four major classes but current state can not switch to adjacent states, system will check current state to determine whether the corresponding pieces of sentence describes the desired information or not. If current state is terminal states, state 4 or state 8, system will output the previous segment that contains candidate items and meets the rules; otherwise the sequence will be ruled out.

We use an example to illustrate how the FSA works. Given a POS tagged sentence: "Overexpression/NN of/IN Myc/NNP induces/VBZ expression/NN of/IN the/DT prohibitions/NNS ./.", the state transition of the POS sequence is 1→ 2→ 1→ 2→ 3→ 4→ 5→ 4. Because of the positions of *Myc *and *prohibitin *in sentence and structure meets the rules defined in FSA, the system will report that *Myc *is related gene of *prohibitin*.

Using this approach described above GeneSum is able to summarize genes according to extracted information of related genes and functions and diseases. And GeneCluster can offer a visualization of functional relationships among genes. Integrating these two modules, GeneLibrarian is a functional screening and information summarization platform that facilitates users to quickly review their interested genes.

## Results

This section investigates the effectiveness of the GeneLibrarian system by summarizing the related gene information and visualizing the annotation result.

### Questing the GeneLibrarian

Users normally retrieve relevant articles by keywords such as genes or other diseases at the PubMed. With GeneLibrarian, users can obtain not only relevant articles, but also a visualized representation of annotation analysis and summarized information of these genes. Before submitting any query about user-specified genes to PubMed, this system organizes them according to annotated semantic similarity computed by the method described above. A well-organized viewgraph will help users to determine the major functions or processes of these genes. Similar work is done by BioRag[9], but it ranks annotations according to the frequency of genes annotated by such terms. In contrast, GeneLibrarian groups them according to their semantic similarity and clusters analogous terms to form warm-colored blocks in the diagonal of an array. For instance, in Figure 1, DNAPK was annotated "double-strand break repair" and ATM, ATR, GADD45, PCNA were annotated "DNA repair". According to the count of genes, these two terms would be separated but they indeed share a similar concept; therefore, our system clustered them properly. In our clustering viewgraph, each warm block represents a major function or process. With the help of the text-mining module, users can expand their gene list with the information extracted from the literature. Specifically, users provide a group of genes, and the GeneLibrarian system summarizes the information of related genes, functions, and diseases. To this information, users can add some of the extracted genes into their list for the next annotation analysis. Moreover, users can also select specific genes and place in a query window whatever terms they want information about. The system will then use these genes and terms to retrieve relevant articles from PubMed and provide users with an absent/present list. Users then can validate the result or any idea inspired by it using these articles.

The GeneLibrarian system offers users not only a convenient platform to gather summarized information about their genes of interest, but also the potential for discovering associated genes, functions, or diseases that may have never been considered.

### Visualizing the GeneCluster

**Figure 6:**
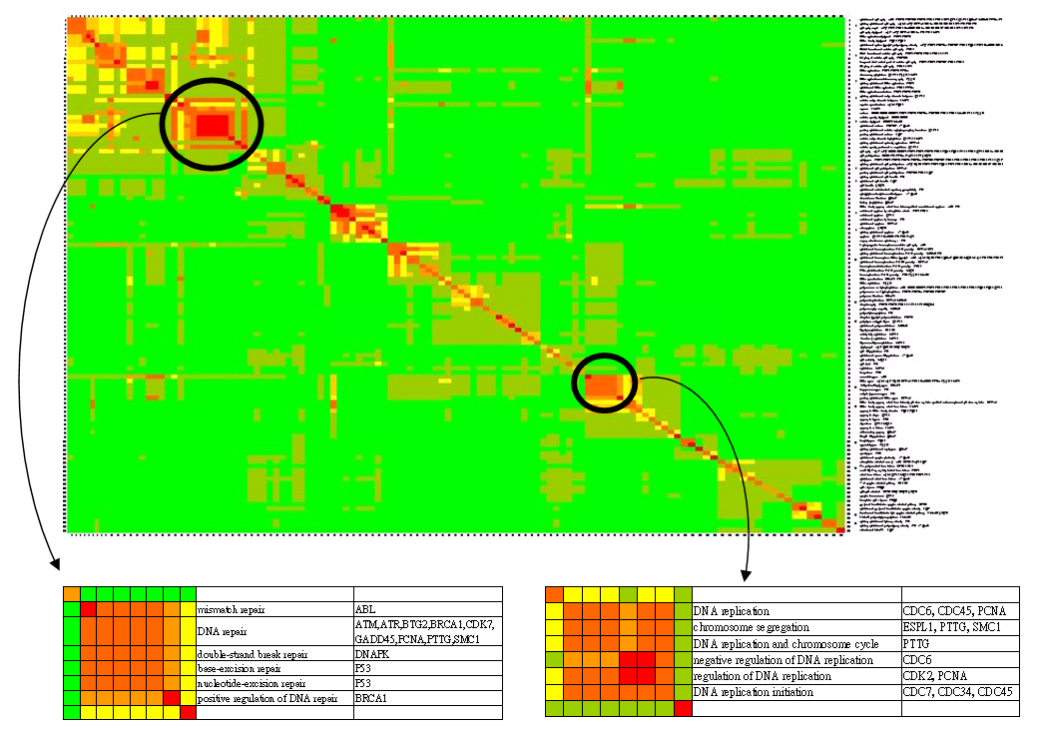
The colorful 2D clustering array in which hotter colors indicate higher similarity, and vice versa.

In order to validate the significance of the resulted viewgraph from our approach, we use human cell cycle related genes set downloaded from the KEGG as the test data. The cell cycle gene list contains 139 genes. We employed the biological process, a recognized series of events or molecular functions, in the GO annotation as the basis for clustering purpose. The GeneCluster produced 2 major clusters and several minor clusters according to the similarity of corresponding annotations, as exhibited in Figure 6. For the two major clusters, we display the degrees of similarity, GO annotations, and corresponding gene names in detail. It can be seen that the 1st and 2nd groups of genes involves two of the most important cellular processes, "DNA repair" and "DNA replication", respectively [10].

**Figure 7:**
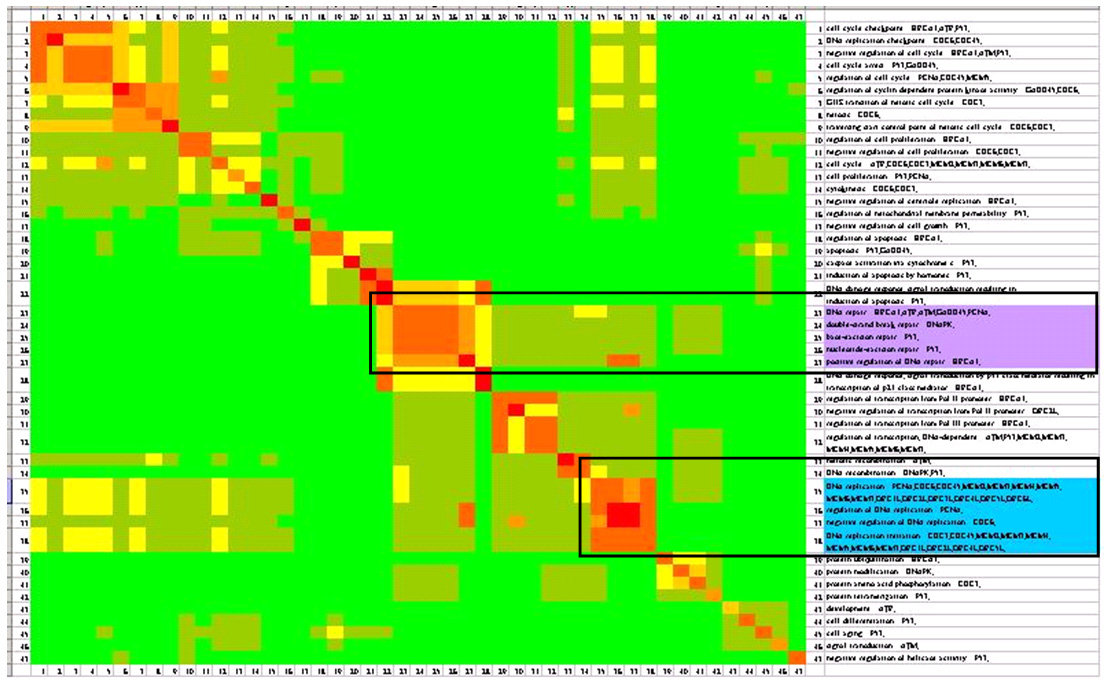
The re-clustering result obtained from the 22 genes involving the "DNA repair" and "DNA replication" cellular processes.

Furthermore, we reconstruct a new clustering viewgraph based on only those 22 genes shown above (7 from DNA repair and 15 from DNA replication). Two distinct clusters were obtained from clustering the annotations again, as illustrated in Figure 7. GeneCluster is able to extract different genes as several groups which with similar molecular function, biological process or cellular location by their Gene Ontology terms. Hopefully users may need to re-cluster repeatedly until they find some interested results in the gene discovery and microarray analysis.

### Evaluating the abilities of information summarization

A convincing well annotated corpus is essential for evaluating performance of a system. But to annotate such corpus manually is not an easy job and it requires domain experts' participation. Three genes, which experts are familiar with, and the related articles were used to evaluate performance of GeneSum. The number of obtained abstracts of prohibitin, TRADD, and TSG101 are 171 and 200 out of 1036 and 189, respectively. The experts annotated these abstracts and examine the results manually, the results are shown in table 1. In GeneSum, we divide the result into two confidence levels: "highly linked" and "linked" for the purpose of providing evidence information to users for reference. The results belonging to former level are more confident than those belonging to later one. Table 2 indicates the ability of GeneSum to extract related information of "highly linked" level from the corpus for those genes mentioned above.

## Discussion and conclusion

In omic era researchers are able to generate a large number of experiment data by many high-throughput techniques such as microarrys. Consequently, how to efficiently review candidate genes is the pressing task that we focus on. In this study, we've developed a platform, GeneLibrarian, which facilitates users to screen functional relationships and summarize their interested group of genes. It is consist of two modules. GeneSum is a text-mining based module, it can summarize genes according to extracted information of related genes and functions and diseases. And the other module is GeneCluster, which are able to offer a visualization of functional relationships among genes. GeneLibrarian concentrates on providing well organized information to users and we believe that will be useful in their researches.

## Availability and requirements

Project name: GeneLibrarian;

The GeneLibrarian is web-accessible at ; Operating system(s): Platform independent;

Programming language: Java;

License: GNU GPL;

Any restrictions to use by non-academics: None.

## Authors' contributions

JHC conceived of the study, participated in its coordination, and drafted the manuscript. JWS participated in benchmark study, and prepared the evaluation materials. CLC designed and implemented prototype of the GeneLibrarian system. HHL refined and improved the system and wrote the manuscript. All authors read and approved the final manuscript.

## Figures and Tables

**Figure 4:**
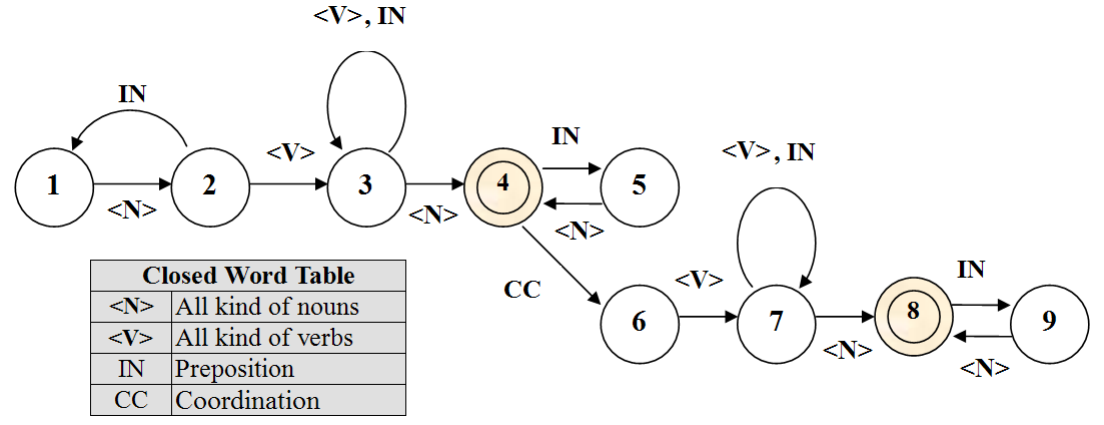
Structure of the finite state automata.

**Figure 5:**
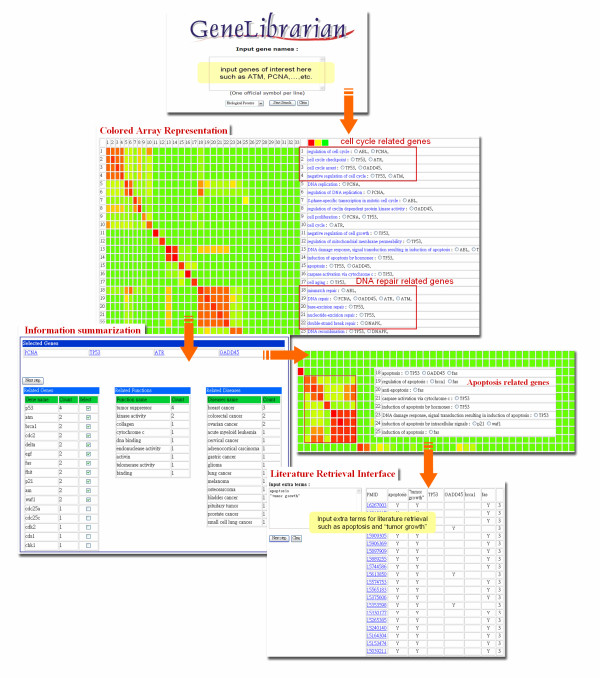
**Questing the GeneLibrarian: illustrative example**. For a given gene list – ABL, ATM, ATR, GADD45, PCNA, DNAPK, and TP53 – the annotation analysis result indicates that there are two distinct processes among these genes: cell cycle and DNA repair. Selecting the queried genes, GeneLibrarian extracts and summarizes related information from the literature, and users can use these summaries to expand their list for further annotation analysis. The result exhibits a group of apoptosis-related genes that users might not think about. Finally, users can enter extra terms like "apoptosis" and "tumor growth"; the system submits these genes and terms to PubMed and lists and ranks PMIDs by the number of the present terms.

**Table 1:** Noun phrase chunking rules for sentences structure simplification

Former POS	Latter POS	Latter not leaded with
JJ	CD	, or .
CD	JJ	, or .
CD	NN	, or .
NN	CD	, or .
NN	NN	, or . or ;
NN	JJ	, or .
JJ	NN	, or .
JJ	JJ	, or .

**Table 2:** Precision rates on evaluation data

Gene names	Summary sentence for related genes	Summary sentence for related functions	Summary sentence for related diseases
*prohibitin*	81.81%	60%	80%
*TRADD*	83.01%	76%	75%
*TSG101*	88.88%	66.66%	88.23%
